# Supplementary material for: Modeling glioblastoma heterogeneity as a dynamic network of cell states
Source: Mol Syst Biol. 2021 Sep 16;17(9):e10105. doi: 10.15252/msb.202010105 (PMC8444284; doi:10.15252/msb.202010105)
Supplement: Supplementary file 6 — Source Data for Figure 5 [file MSB-17-e10105-s004.zip › Figure5A_sourcedata/GSEA_3071/hallmarks_stateV.GseaPreranked.1623742546329/HALLMARK_UNFOLDED_PROTEIN_RESPONSE.html]

Details for gene set HALLMARK\_UNFOLDED\_PROTEIN\_RESPONSE[GSEA]

|  || Dataset | state43071 |
| Phenotype | NoPhenotypeAvailable |
| Upregulated in class | na\_neg |
| GeneSet | HALLMARK\_UNFOLDED\_PROTEIN\_RESPONSE |
| Enrichment Score (ES) | -0.41592643 |
| Normalized Enrichment Score (NES) | -1.9948828 |
| Nominal p-value | 0.0 |
| FDR q-value | 0.002287066 |
| FWER p-Value | 0.022 |
Table: GSEA Results Summary

  

Fig 1: Enrichment plot: HALLMARK\_UNFOLDED\_PROTEIN\_RESPONSE      
 Profile of the Running ES Score & Positions of GeneSet Members on the Rank Ordered List

  

| PROBE | GENE SYMBOL | GENE\_TITLE | RANK IN GENE LIST | RANK METRIC SCORE | RUNNING ES | CORE ENRICHMENT || 1 | FUS |  |  | 590 | 0.806 | -0.0844 | No |
| 2 | DCP2 |  |  | 762 | 0.717 | -0.0920 | No |
| 3 | VEGFA |  |  | 926 | 0.649 | -0.1004 | No |
| 4 | LSM4 |  |  | 1003 | 0.619 | -0.0933 | No |
| 5 | EXOSC9 |  |  | 1472 | 0.487 | -0.1656 | No |
| 6 | CKS1B |  |  | 1502 | 0.479 | -0.1544 | No |
| 7 | SPCS1 |  |  | 1778 | 0.422 | -0.1921 | No |
| 8 | DCP1A |  |  | 2093 | 0.374 | -0.2389 | No |
| 9 | NFYB |  |  | 2330 | 0.344 | -0.2719 | No |
| 10 | CXXC1 |  |  | 2619 | 0.308 | -0.3161 | No |
| 11 | CNOT6 |  |  | 2679 | 0.302 | -0.3168 | No |
| 12 | TATDN2 |  |  | 2892 | 0.280 | -0.3475 | No |
| 13 | PDIA6 |  |  | 3003 | 0.269 | -0.3591 | No |
| 14 | PARN |  |  | 3085 | 0.261 | -0.3654 | No |
| 15 | NOP14 |  |  | 3247 | -0.259 | -0.3871 | No |
| 16 | ARFGAP1 |  |  | 3361 | -0.276 | -0.3990 | No |
| 17 | SLC30A5 |  |  | 3384 | -0.279 | -0.3935 | No |
| 18 | SPCS3 |  |  | 3415 | -0.284 | -0.3893 | No |
| 19 | TTC37 |  |  | 3460 | -0.292 | -0.3875 | No |
| 20 | ERO1A |  |  | 3506 | -0.300 | -0.3856 | No |
| 21 | GOSR2 |  |  | 3535 | -0.305 | -0.3803 | No |
| 22 | SDAD1 |  |  | 3702 | -0.332 | -0.4004 | No |
| 23 | DNAJB9 |  |  | 3721 | -0.334 | -0.3922 | No |
| 24 | PSAT1 |  |  | 3732 | -0.336 | -0.3824 | No |
| 25 | EIF2S1 |  |  | 3809 | -0.351 | -0.3846 | No |
| 26 | HSPA9 |  |  | 3909 | -0.369 | -0.3907 | No |
| 27 | EEF2 |  |  | 3913 | -0.369 | -0.3784 | No |
| 28 | EXOC2 |  |  | 3973 | -0.383 | -0.3763 | No |
| 29 | DDIT4 |  |  | 4182 | -0.432 | -0.4009 | Yes |
| 30 | EIF4A1 |  |  | 4210 | -0.442 | -0.3906 | Yes |
| 31 | MTHFD2 |  |  | 4212 | -0.444 | -0.3753 | Yes |
| 32 | STC2 |  |  | 4358 | -0.488 | -0.3859 | Yes |
| 33 | SEC11A |  |  | 4406 | -0.503 | -0.3774 | Yes |
| 34 | IMP3 |  |  | 4477 | -0.529 | -0.3723 | Yes |
| 35 | ATF4 |  |  | 4509 | -0.545 | -0.3592 | Yes |
| 36 | SLC7A5 |  |  | 4513 | -0.547 | -0.3407 | Yes |
| 37 | XPOT |  |  | 4596 | -0.586 | -0.3359 | Yes |
| 38 | WIPI1 |  |  | 4599 | -0.589 | -0.3158 | Yes |
| 39 | HSPA5 |  |  | 4714 | -0.652 | -0.3148 | Yes |
| 40 | RPS14 |  |  | 4715 | -0.653 | -0.2920 | Yes |
| 41 | HERPUD1 |  |  | 4762 | -0.687 | -0.2768 | Yes |
| 42 | SEC31A |  |  | 4924 | -0.835 | -0.2784 | Yes |
| 43 | FKBP14 |  |  | 4954 | -0.872 | -0.2535 | Yes |
| 44 | KDELR3 |  |  | 4974 | -0.893 | -0.2260 | Yes |
| 45 | YIF1A |  |  | 5015 | -0.959 | -0.2002 | Yes |
| 46 | NPM1 |  |  | 5040 | -1.017 | -0.1694 | Yes |
| 47 | DDX10 |  |  | 5061 | -1.053 | -0.1364 | Yes |
| 48 | CEBPB |  |  | 5076 | -1.088 | -0.1012 | Yes |
| 49 | SHC1 |  |  | 5094 | -1.133 | -0.0649 | Yes |
| 50 | XBP1 |  |  | 5165 | -1.393 | -0.0297 | Yes |
| 51 | NABP1 |  |  | 5203 | -1.558 | 0.0175 | Yes |
Table: GSEA details [plain text format]

  

Fig 2: HALLMARK\_UNFOLDED\_PROTEIN\_RESPONSE: Random ES distribution      
 Gene set null distribution of ES for **HALLMARK\_UNFOLDED\_PROTEIN\_RESPONSE**
